# On the Origin and Evolutionary Relationships of the Reverse Transcriptases Associated With Type III CRISPR-Cas Systems

**DOI:** 10.3389/fmicb.2018.01317

**Published:** 2018-06-15

**Authors:** Nicolás Toro, Francisco Martínez-Abarca, Alejandro González-Delgado, Mario Rodríguez Mestre

**Affiliations:** Structure, Dynamics and Function of Rhizobacterial Genomes, Grupo de Ecología Genética de la Rizosfera, Department of Soil Microbiology and Symbiotic Systems, Estación Experimental del Zaidín, Consejo Superior de Investigaciones Científicas, Granada, Spain

**Keywords:** Cas1, CRISPR-Cas system, genome evolution, group II introns, retroelements, reverse transcriptase, ribozymes

## Abstract

Reverse transcriptases (RTs) closely related to those encoded by group II introns but lacking the intron RNA structure have been found associated with type III clustered regularly interspaced short palindromic repeats (CRISPR)-Cas systems, a prokaryotic immune system against invading viruses and foreign genetic elements. Two models have been proposed to explain the origin and evolutionary relationships of these RTs: (i) the “single point of origin” model, according to which these RTs originated from a single acquisition event in bacterial, with the various protein domains (RT, RT-Cas1, and Cas6-RT-Cas1 fusions) corresponding to single points in evolution; and (ii) the “various origins” model, according to which, independent acquisition events in different evolutionary episodes led to these fusions. We tested these alternative hypotheses, by analyzing and integrating published datasets of RT sequences associated with CRISPR-Cas systems and inferring phylogenetic trees by maximum likelihood (ML) methods. The RTs studied could be grouped into 13 clades, mostly in bacteria, in which they probably evolved. The various clades appear to form three independent lineages in bacteria and a recent lineage in archaea. Our data show that the Cas6 domain was acquired twice, independently, through RT-Cas1 fusion, in the bacterial lineages. Taken together, there more evidence to support the “various origins” hypothesis.

## Introduction

Phylogenetic analyses have shown that bacterial Reverse transcriptases (RTs) can be classified into 17 main groups, and that over 50% of these enzymes, which can be used to generate complementary DNA (cDNA) from an RNA template, are encoded by group II introns (Toro and Nisa-Martínez, 2014; Zimmerly and Wu, 2015). These introns act as ribozymes and mobile retroelements (Michel et al., 1989; Ferat and Michel, 1993; Toro et al., 2007; Lambowitz and Zimmerly, 2011). Group II intron-encoded RTs can be classified into several major groups (Simon et al., 2008; Toro and Martínez-Abarca, 2013): A, B, C, D, E, F, G (formerly *g1*), CL1 (chloroplast-like 1), CL2 (chloroplast-like 2), and ML (mitochondrion-like). These retroelements may have evolved in Eubacteria, through the association of an ancient RT with a structured catalytic RNA to form the ancestral group II intron (Toro et al., 2007; Zimmerly and Semper, 2015). Interestingly, RTs closely related to those encoded by group II introns but lacking a recognizable intron RNA structure have been identified, and some of these RTs are associated with type III clustered regularly interspaced short palindromic repeats (CRISPR)-Cas systems, adjacent or fused at the C-terminus to Cas1 (Kojima and Kanehisa, 2008; Simon and Zimmerly, 2008; Toro and Nisa-Martínez, 2014; Silas et al., 2016, 2017a; Toro et al., 2017; Shmakov et al., 2018). CRISPR-Cas is a complex system of defense that provides adaptive immunity against invading viruses and plasmids by sequence specific targeting of nucleic acids (Horvath and Barrangou, 2010). Foreign DNA is recognized and cleavage by Cas1 and Cas2 proteins generating novel DNA spacers that are incorporated into the CRISPR array (adaptation), which is transcribed into a pre-crRNA (precursor transcript) that is processed into mature crRNA, which are used as a guide by a Cas complex (effector complexes) that recognize and bind the complementary invading nucleic acid, resulting in degradation of the target molecule (interference). Attention has recently focused on these RTs associated with CRISPR-Cas systems because, in a CRISPR-Cas type III-B displaying transcription-dependent DNA interference (Silas et al., 2017b) harbored by the marine bacterium *Marinomonas mediterranea* (MMB-1), the associated RT-Cas1 fusion has been shown to facilitate the RT-dependent acquisition of RNA spacers *in vivo* through a mechanism resembling group II intron retrohoming (Silas et al., 2016).

The evolutionary origin of such associations of an RT with a type III CRISPR-Cas system and their phylogenetic relationships remain a mystery. Based on the topology of a maximum likelihood phylogenetic tree reconstructed from 134 RT sequences associated with CRISPR-*cas* loci obtained with the *FastTree* (Price et al., 2010) program, Silas et al. (2017a) recently suggested a parsimonious evolutionary scenario known as the “single point of origin” model. According to this model, an RT first arrives at a CRISPR-Cas adaptation module, possibly through a random group II intron retrotransposition event. The C-terminus of this RT is then fused to the adjacent Cas1, and a Cas6 domain is then acquired at the N-terminus (Silas et al., 2017a). However, two cases of independent RT acquisitions by CRISPR-*cas* loci have been identified: one in the archaeal genus *Methanosarcina* and the other associated with type I-E CRISPR-Cas systems (Silas et al., 2017a; for a review see Koonin and Makarova, 2017). The first of these acquisitions was probably triggered by a group II intron retrotransposition event, whereas the second may have involved RT acquisition from a type III CRISPR-*cas* locus. Almost concomitantly, we used *FastTree* to reconstruct a phylogenetic tree from 118 RTs associated with CRISPR-Cas systems and reported that these RTs could be grouped into 12 major clades, suggesting that the association of an RT with a CRISPR-Cas system may have occurred on numerous occasions during evolution (Toro et al., 2017). In this report, we attempt to shed light on the two scenarios proposed to explain the origin and evolutionary relationships of the RTs associated with CRISPR-Cas systems, by analyzing the branches and clades identified in these previous studies, and performing a new phylogenetic analysis on the integrated dataset.

## Materials and Methods

### Phylogenic Analyses of RT Sequences

**FIGURE 1:**
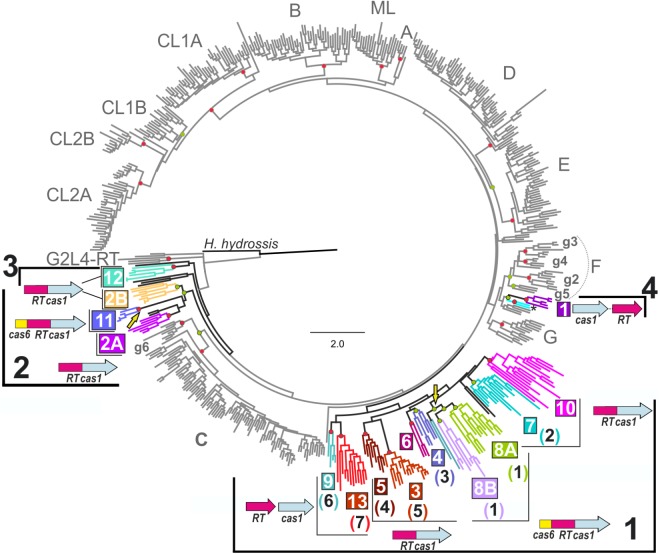
Phylogenetic tree of Reverse transcriptases (RTs) associated with clustered regularly interspaced short palindromic repeats (CRISPR)-Cas systems. The tree was inferred with *FastTree*, from an alignment containing 558 RT sequences, including 137 RT sequences closely related to group II intron-encoded RTs associated with CRISPR-Cas systems (see section “Materials and Methods”). Group II intron classes and varieties are indicated in gray, together with the RTs of the G2L4 group. Branches corresponding to RTs associated with CRISPR-*cas* loci are in shown in black and the clades and host bacterial phyla or archaeal family are indicated by highlighting in color. The possible bacterial lineages 1, 2 and 3, and archaeal lineage 4 are indicated. The two colored branches below clade 1 correspond to two unique RT sequences related to the archaeal RTs of clade 1. The most common domain or gene organization for each clade is indicated. Independent genes are shown with distinct arrows, while fused genes are displayed as single arrows with multiple colors. The name of the clade (colored and boxed) and its correspondence with the branches (number in brackets) identified by Silas et al. (2017a) are shown. Yellow arrows indicate two independent Cas6 domain acquisitions. Circles at the nodes indicate that the node concerned has a FastTree local support value ≥ 0.9 and either a standard non-parametric bootstrap value ≥ 70% (Red) or SH-aLRT and Ufboot values ≥ 80% (Green) in the corresponding phylogenetic analyses. *Haliscomenobacter hydrossis* (GI: 332661943) corresponds to a retron/retron-like RT, and was used as the outgroup.

We used MUSCLE software (Edgar, 2004) to align (at 250 positions) 21 RT-like sequences from the identified branches 7, 8, and 9 reported by Silas et al. (2017a) absent from our previous study against our dataset (Toro et al., 2017) comprising 537 sequences and encompassing RT domains (0–7) including 414 sequences of the known group II intron RT classes, three RTs from the closely related G2L4 group (Toro and Nisa-Martínez, 2014) with no recognizable group II intron structure, two RT sequences related to the archaeal RTs associated with CRISPR-Cas systems of clade 1, and 118 RT sequences associated with CRISPR-*cas* loci. This last group of sequences included a group II intron of the ML class (*Herpetosiphon aurantiacus* GI: 159898445) and a retron-like RT sequence (*Haliscomenobacter hydrossis*, GI: 332661943). The 137 RT sequences closely related to group II intron-encoded RTs associated with CRISPR-Cas systems used for the phylogenetic analyses are presented in **Supplementary Table S1**. A phylogenetic tree (**Figure 1**) was constructed with the *FastTree* program (Price et al., 2010) as previously described (Toro et al., 2017). *IQ-TREE* v. 1.6.1 (Nguyen et al., 2015) was also used to infer phylogenetic trees (**Supplementary Figure S1**) from the above alignment, using the amino-acid substitution best-fit model (LG+G4) provided by ModelFinder selected on the basis of the Bayesian information criterion (BIC). Branch support was assessed by ultrafast bootstrap approximation (UFBoot), and the impact of severe model violations was reduced by using hill-climbing nearest interchange (NNI) search, SH (Shimodaira-Hasegawa)-aLRT (approximate likelihood ratio test, Guindon et al., 2010) with 1000 replicates, and standard non-parametric bootstrap (100 replicates). The retron-like RT associated with a CRISPR-Cas system from *H. hydrossis* was placed at the root of the tree as an outgroup. In the FastTree phylogenetic tree shown in **Figure 1**, the clades were assigned to the inner nodes with FastTree support values ≥ 0.9 and if they had a standard non-parametric bootstrap value ≥ 70% or SH-aLRT and Ufboot values ≥ 80% in the other trees.

## Results and Discussion

A comparison of the RT datasets used for phylogenetic tree reconstruction by Silas et al. (2017a) and Toro et al. (2017) highlighted several differences, most of which could be explained by the different compilation methods used to retrieve RT-like sequences associated with CRISPR-*cas* loci. Thus, the RTs separated from Cas1 clustered on branches 7 and 10 in the article by Silas et al. (2017a) were not included in Toro et al. (2017), and conversely, the RTs grouped into clade 11 and fused to Cas1, carrying a Cas6 domain at their N-termini, were not included in Silas et al. (2017a). Similarly, clades 6 and 10 were not resolved by Silas et al. (2017a), because the reported tree contained only a few members of these clades, which thus appeared as unclassified entries. Silas et al. (2017a) reported a group at the base of the tree (branch 10) with RT-like sequences that appeared to be separated from the adjacent Cas1 sequence. This group of sequences was characteristic of *Streptomyces* and *Streptococcus* species and, remarkably, associated with type I-E CRISPR-*cas* loci. We noticed that these putative RTs had no conserved group II intron-like maturase domain, and preliminary phylogenetic analyses (not shown) with a dataset of 742 RT sequences (Toro and Nisa-Martínez, 2014) indicated that the RT-like sequence from *Streptococcus oralis* SK10 belonged to the uncharacterized UG2 group of RTs, whereas those from *Streptomyces clavuligerus* ATCC27064, *Streptomyces lydicus* A02 and *Streptomyces* MUSC164 formed a phylogenetically distant clade to group II intron classes. These last three RT sequences had a large number of substitutions per site (FastTree: 3.2), a WGDD sequence in place of the canonical YADD sequence in RT domain 5, and they lacked the conserved domains RT0 and 7. The *Streptococcus* and *Streptomyces* RT-like sequences described above do not, therefore, have a common ancestor, and they appear to be only distantly related to group II intron-encoded RTs. They will not, therefore, be considered further here.

We investigated the origin and evolutionary relationships of the RTs associated with type III CRISPR-Cas systems further, by aligning (250 positions) the RT sequences (see section “Materials and Methods” and **Supplementary Table S1**) from the study by Silas et al. (2017b) absent from our previous study (Toro et al., 2017) with the 537 sequences of our dataset. The inferred phylogenetic tree reconstructed for the integrated 558 RT sequences with *FastTree* is shown in **Figure 1**. The *IQ-TREE* program (Nguyen et al., 2015) was also used to infer phylogenetic trees from the above alignment (see section “Materials and Methods”). Together, these analyses provided additional support for the previously identified 12 clades of RTs associated with CRISPR-Cas systems, and confirmed the existence of branch 7 as described by Silas et al. (2017a), hereafter referred to as clade 13. Most of the sequences that Silas et al. (2017a) clustered together on branch 9 were grouped into clades 2A (*Caminibacter mediatlanticus* TB-2), and 2B (*Bacteroides fragilis* str. 3988T B14 and *Bacteroides barnesiae* DSM 18169) (see below). By contrast, the minor branch 8 identified by Silas et al. (2017a) was not confirmed in our analyses, even though the *Roseburia inulivorans* DSM 16841 RT clustered within our clade 12 (phylum Firmicutes). Other RTs from this hypothetical branch remained unclassified (**Supplementary Table S1**) and may have a distinct origin. The clades identified in our previous work (Toro et al., 2017), in this study (clades 1–13), and the equivalent branches identified by Silas et al. (2017a) are shown in **Figure 1**.

The incorporation of additional RT sequences into our dataset led to the sequences clustered in clade 11 (Cas6-RT-Cas1 fusions) branching off from a well-supported node (FastTree local value 0.98, and SH-aLRT and Ufboot values of 94 and 94%, respectively) common to sequences formerly clustered in clade 2 (RT-Cas1 fusions), hereafter referred to as 2/11. Likewise, the RT sequences previously clustered in clade 2, mostly belonging to the new proposed phylum Epsilonbacteraeota, comprising the Epsilonproteobacteria and Desulfurellales (phyl. Nov., Waite et al., 2017) and the phylum Bacteroidetes, could be split into two subclades, 2A and 2B, respectively. Interestingly, the RTs of clade 11 were also found to belong to phylum Bacteroidetes. The close relationships between clades 2 and 11 were not unexpected, because phylogenetic analyses of associated and co-evolving Cas1 proteins had already indicated that the two clades might have a common ancestor (Toro et al., 2017). Thus, the phylogenetic analyses suggest that a Cas6 domain was acquired by the ancestor of some *Porphyromonas* species (clade 11), via a preexisting RT-Cas1 fusion within the phylum Bacteroidetes (clade 2B). The acquisition this protein domain in clade 11 therefore constitutes an independent evolutionary event similar to the acquisition that occurred in clade 8 (**Figure 1**), arguing against the “single point of origin” scenario. Clade 12 (RT-Cas1 fusions) restricted to the phylum Firmicutes, in all phylogenetic analyses is placed at the base of the tree (**Figure 1** and **Supplementary Figure S1**), and it would appear as an independent lineage from the other clades.

*FastTree* also provided support for an inner node comprising clades 3–10, and 13 (local value of 0.96), consistent with the “single point of origin” hypothesis: acquisition of an RT (clades 9 and 13), followed by a fusion of the RT to Cas1 (clades 3–7 and 10) and, finally, the acquisition of a Cas6 domain by a RT-Cas1 fusion (clade 8). However, this node was not consistently supported by other ML methods (SH-aLRT and Ufboot values of 53.5 and 67%, respectively), and clades 9 and 13 split (RTs not fused to Cas1) from a different node to the other clades in the non-parametric bootstrapping analysis (**Supplementary Figure S1**). Further studies are therefore required to confirm or rule out these relationships.

**FIGURE 2:**
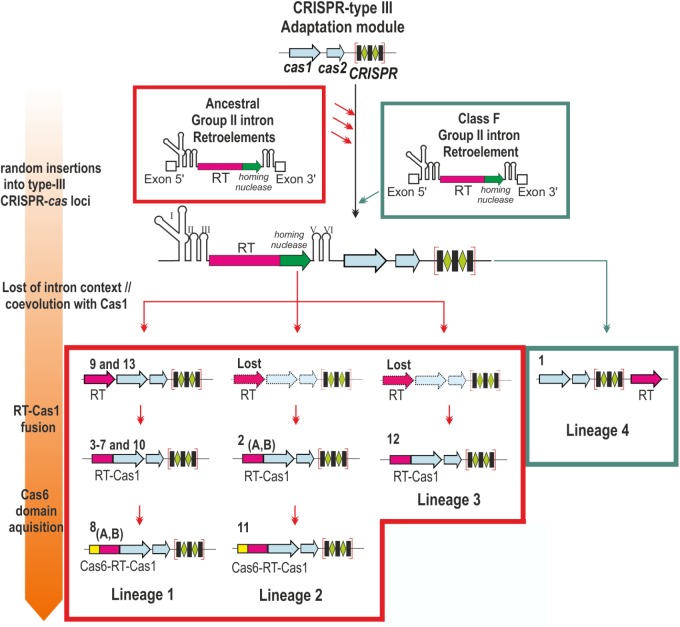
Contribution of group II introns to the origin of the reverse transcriptases associated with type III CRISPR-Cas systems. The scheme represents the different stages (described at Left) in the association of a group II intron-encoded RT to CRISPR type III loci. Distinct CRISPR-*cas* loci were independently invaded by ancestral group II introns and a more recent Class F intron (red and green arrows, respectively). The intron RNA component was lost and the remaining RT coevolved with the adjacent Cas1 protein, a process that may have occurred independently four times during evolution (Lineages 1–4). Subsequently the RT and Cas1 were fused (Lineages 1–3), and later a Cas6 domain was acquired independently twice (Lineages 1 and 2). The clades and their corresponding RT-gene organization are indicated. Dashed gene loci indicates RT arrangements not yet identified (Lost).

Most of the internal nodes lacked reliable support, due to the long diversification time and sequence saturation with mutations, hindering inferences about the relationships between group II intron classes and the distinct CRISPR-Cas/RT clades. However, the topology of the trees reflected the closer relationship of the clades, with the exception of clade 1 (hereafter referred to as lineage 4), to class C introns and their possible subdivision into three bacterial lineages: a large lineage comprising clades 3–10 and 13 (lineage 1), the clade 2/11 (lineage 2), and clade 12 (lineage 3). These lineages seem to correspond to acquisitions preceding that in the minor lineage 4 in archaea, possibly from ancestral group II introns (**Figure 2**).

## Conclusion

Our results suggest that RTs may have become associated with CRISPR-Cas systems on various occasions: (i) the identified clades do not all branch off from a single supported node, and there is, therefore, no consistent evidence in favor of their monophyly; (ii) according to their phylogenetic positions relative to group II intron classes, they appear to form three major lineages in bacteria and one minor lineage in archaea; (iii) two clades including Cas6-RT-Cas1 fusions (clades 8 and 11) branching off at different positions and do not, therefore, appear to have a common ancestor; (iv) the archaeal RT associated with CRISPR-Cas systems (clade 1) appears to have evolved more recently, suggesting that this association may often occur as the result of group II intron retrotransposition events, and (v) some RTs associated with CRISPR-Cas system are only distantly related to group II intron-encoded RTs (i.e., retron-like and others of unknown origin). The phylogeny of these RTs is not fully resolved, and further extensive studies should therefore provide more insight into their origin and evolutionary relationships.

## Author Contributions

NT wrote the manuscript with input from FM-A and AG-D. MRM contributes to RT sequences analyses. All the authors critically reviewed the manuscript.

## Conflict of Interest Statement

The authors declare that the research was conducted in the absence of any commercial or financial relationships that could be construed as a potential conflict of interest.

## Supplementary Material

FIGURE S1 The inferred phylogenetic trees inferred with FastTree **(A)**, and IQ-Tree using SH-aLRT/Ufboot or non-parametric bootstrapping (**B,C**, respectively) are represented. With the exception of group II introns of class C and the variety *g6* the other intron classes were collapsed for the sake of simplicity. The 13 identified clades of RTs associated to type III CRISPR-Cas systems are indicated in color. G2L4 is a group of RTs that lack the intron RNA structure and are not associated to CRISPR-Cas systems.

TABLE S1 List of 137 unique Reverse transcriptase (RT) sequences associated with CRISPR-Cas systems used in **Figure 1**.
