# Supplementary figures and images for: On the Origin and Evolutionary Relationships of the Reverse Transcriptases Associated With Type III CRISPR-Cas Systems
Source: Front Microbiol. 2018 Jun 15;9:1317. doi: 10.3389/fmicb.2018.01317 (PMC6013744; doi:10.3389/fmicb.2018.01317)

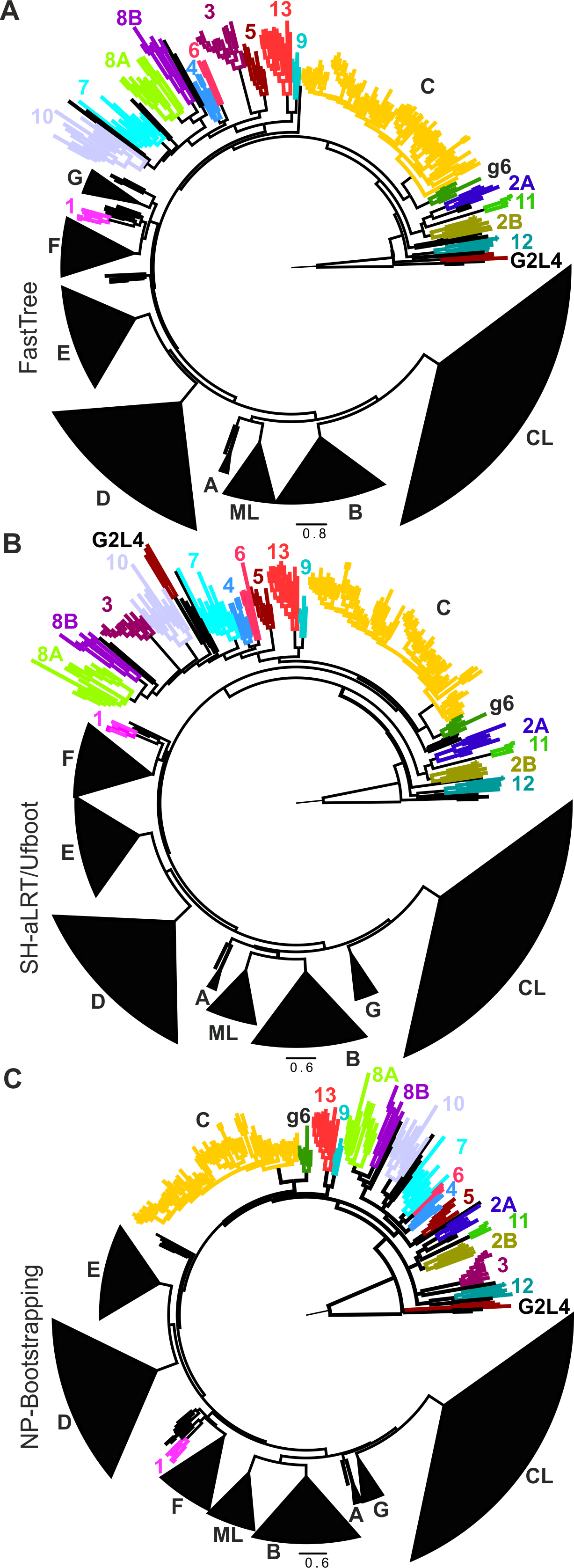

Supplement: FIGURE S1 — The inferred phylogenetic trees inferred with FastTree (A), and IQ-Tree using SH-aLRT/Ufboot or non-parametric bootstrapping (B,C, respectively) are represented. With the exception of group II introns of class C and the variety g6 the other intron classes were collapsed for the sake of simplicity. The 13 identified clades of RTs associated to type III CRISPR-Cas systems are indicated in color. G2L4 is a group of RTs that lack the intron RNA structure and are not associated to CRISPR-Cas systems. [file Image_1.TIF]
